# Expression of Npas4 mRNA in Telencephalic Areas of Adult and Postnatal Mouse Brain

**DOI:** 10.3389/fnana.2015.00145

**Published:** 2015-11-18

**Authors:** Joanne C. Damborsky, G. Simona Slaton, Ursula H. Winzer-Serhan

**Affiliations:** Department of Neuroscience and Experimental Therapeutics, Texas A&M University System Health Science CenterBryan, TX, USA

**Keywords:** telencephalon, LE-PAS, cortex, hippocampus, development, *in situ* hybridization, synaptogenesis

## Abstract

The transcription factor neuronal PAS domain-containing protein 4 (Npas4) is an inducible immediate early gene which regulates the formation of inhibitory synapses, and could have a significant regulatory role during cortical circuit formation. However, little is known about basal Npas4 mRNA expression during postnatal development. Here, postnatal and adult mouse brain sections were processed for isotopic *in situ* hybridization using an Npas4 specific cRNA antisense probe. In adults, Npas4 mRNA was found in the telencephalon with very restricted or no expression in diencephalon or mesencephalon. In most telencephalic areas, including the anterior olfactory nucleus (AON), piriform cortex, neocortex, hippocampus, dorsal caudate putamen (CPu), septum and basolateral amygdala nucleus (BLA), basal Npas4 expression was detected in scattered cells which exhibited strong hybridization signal. In embryonic and neonatal brain sections, Npas4 mRNA expression signals were very low. Starting at postnatal day 5 (P5), transcripts for Npas4 were detected in the AON, CPu and piriform cortex. At P8, additional Npas4 hybridization was found in CA1 and CA3 pyramidal layer, and in primary motor cortex. By P13, robust mRNA expression was located in layers IV and VI of all sensory cortices, frontal cortex and cingulate cortex. After onset of expression, postnatal spatial mRNA distribution was similar to that in adults, with the exception of the CPu, where Npas4 transcripts became gradually restricted to the most dorsal part. In conclusion, the spatial distribution of Npas4 mRNA is mostly restricted to telencephalic areas, and the temporal expression increases with developmental age during postnatal development, which seem to correlate with the onset of activity-driven excitatory transmission.

## Introduction

Npas4 is a member of the PAS family of proteins, a family of signal transduction molecules characterized by a conserved basic-helix-loop-helix motif and pas domain. Pas family proteins regulate a variety of biologically critical pathways, including those that are important for responses to external stimuli in adults and during development (Gu et al., 2000). In the brain, Npas4 acts as an early-response transcription factor that, when induced by excitatory neuronal activity, regulates the formation of inhibitory synapses onto excitatory and inhibitory neurons (Lin et al., 2008; Coutellier et al., 2012; Bloodgood et al., 2013). By regulating inhibitory synapse numbers, Npas4 can affect the excitatory-inhibitory balance within neural circuits (Spiegel et al., 2014). Imbalances in excitatory and inhibitory activity have been implicated in several neurological disorders (Gao and Penzes, 2015), and thus, Npas4 may be a candidate gene for conditions such as anxiety, autism, bipolar depression and cognitive disorders, which often manifest during development (Jaehne et al., 2015).

In the adult mammalian brain, Npas4 mRNA is found in limbic structures, cortex, and striatum (Moser et al., 2004; Ooe et al., 2004; Shamloo et al., 2006). Although basal brain expression is low, it is rapidly upregulated in response to stimuli causing intense excitatory activity suggesting a role in cortical plasticity (Flood et al., 2004; Shamloo et al., 2006; Ploski et al., 2010; Bloodgood et al., 2013; Kaliszewska and Kossut, 2015). Npas4 is also upregulated in response to acute stress (Drouet et al., 2015), but in contrast, is down-regulated after chronic restraint stress or light deprivation (Yun et al., 2010; Karpova et al., 2010).

During development, activity-dependent synapse formation is critically important, especially during periods when sensory experience shapes cortical circuits (Hensch, 2005). In rodent cortical structures, inhibitory synapses begin to form during late prenatal and early postnatal development (Leinekugel, 2003), and are fine-tuned by neuronal activity (Chattopadhyaya et al., 2004). Despite the potentially important role of Npas4 in establishing inhibitory synapses, little is known about the spatial and temporal expression of Npas4 in the postnatal mammalian brain. In this study, using highly sensitive isotopic *in situ* hybridization, we determined the expression pattern of Npas4 mRNA in postnatal and adult mouse brain under basal, non-challenged conditions.

## Materials and Methods

### Animals and Tissue Preparation

All animals were handled and housed in accordance with the rules stipulated by the Texas A&M University Animal Care Committee. C57BL/6 mice were housed in an animal care facility at 22–25°C with a 12-h light/dark cycle and *ad libitum* food and water. Dams were allowed to give birth in the Texas A&M University vivarium, and pups were nursed until postnatal day 21 (P21). Both male and female pups were used for experimentation but were not sexed for experiments. Only male mice were used at P60. At postnatal days (P)1, 3, 5, 8, 13, 21, and 60 (young adult), pups and adults were brought to the lab in the morning, decapitated and their brains rapidly removed. Whole brains were immediately submerged in isopentane held at −20°C for 30 s, then stored at −80°C. 20 μm coronal sections were taken through the brain using a Microtome cryostat (MICROM International GmbH) kept at −20°C, and thaw-mounted on slides coated in poly-L-lysine (Sigma Chemical, St. Louis, MO, USA). Slides were fixed in 6% formaldehyde in 0.1 M Phosphate Buffer solution (PB), then rinsed twice in 0.1 M PB, once in ddH_2_0, dried and stored at −20°C.

### *In situ* Hybridization

An ^35^S-UTP-labeled cRNA probe for Npas4 was synthesized using a 762 bp. cDNA template, which was constructed via RT-PCR using forward (AGT GGC AGC ACT ACC TGG ATT TCT) and reverse (TCA GAG TTT AGC TGC TGG CGA AGA) primers (Integrated DNA Technologies), resulting in a final PCR product length of 762 bp. The PCR product was subcloned into a pPCR-Script AMP SK (+) plasmid (Stratagene, La Jolla, CA, USA). Sequencing results verified the correct sequence for mouse Npas4 mRNA. The plasmid was linearized via restriction enzyme digest, and cRNA probes were synthesized in the sense and antisense orientation with T3 and T7 RNA polymerase, respectively (Ambion, Austin, TX, USA) in the presence of ^35^S-UTP (PerkinElmer, Boston, MA, USA). Non-specific hybridization was determined with the sense probe, and showed no or negligible hybridization signal (Figure, 1A insert). Specific hybridization was detected with the anti-sense probe and showed the expected expression pattern in adult mouse brain sections.

**Figure 1:**
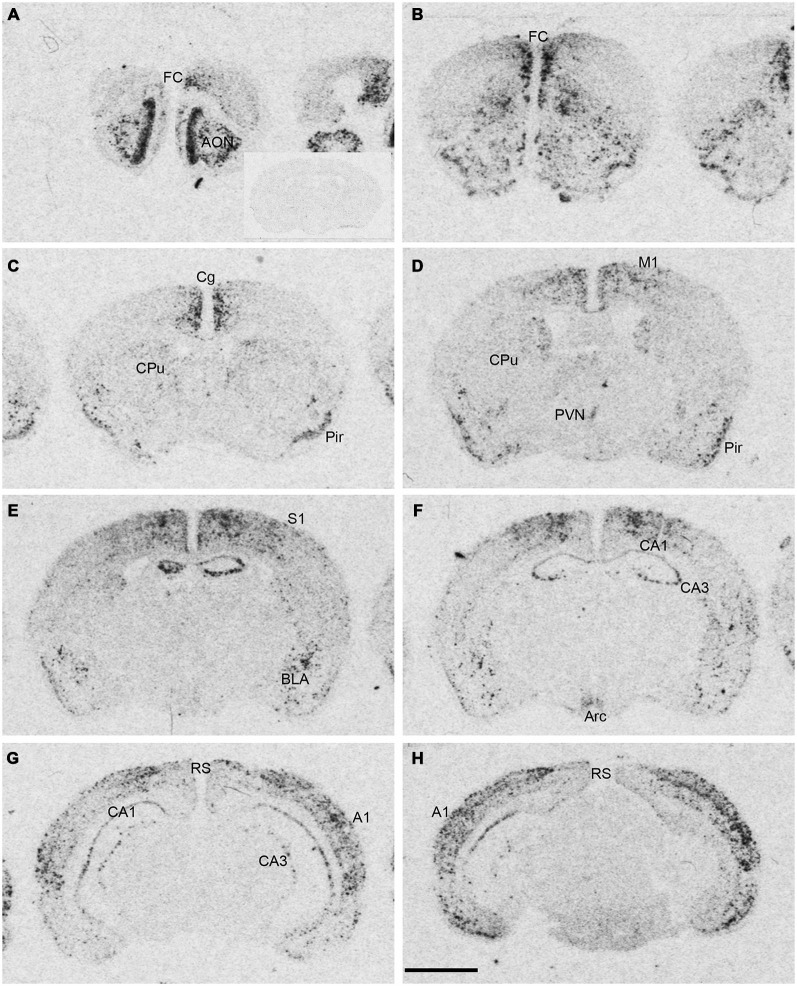
**Npas4 mRNA expression in adult mouse brains.** Autoradiographic images of Npas4 mRNA hybridization in coronal sections of **(A,B)** the anterior olfactory nucleus (AON) and frontal cortex (FC), **(C,D)** caudate putamen (CPu), **(E,F)** dorsal hippocampus, **(G,H)** ventral hippocampus. Insert in **(A)** represents an adult mouse brain section at the level of the dorsal hippocampus hybridized with the Npas4 sense probe. Abbreviations: A1, primary auditory cortex; AON, anterior olfactory nucleus; Arc, arcuate nucleus; BLA, basolateral amygdala nucleus; Cg, cingulate cortex; DG, dentate gyrus; FC, frontal cortex; M1, primary motor cortex; Pir, piriform cortex; PVN, paraventricular hypothalamic nucleus; RS, retrosplenial cortex; S1 primary somatosensory cortex; V1, primary visual cortex. Scale bar = 2 mm.

*In situ* hybridization was performed as previously described (Winzer-Serhan et al., 1999). Briefly, slides containing brain slices were pretreated for 10 min with protease K (0.1 μg/mL), acetylated, dehydrated in graded ethanols and dried in a cold air stream. Slides were then incubated overnight at 60°C with the ^35^S-UTP-labeled cRNA probe in hybridization solution (50% formamide, 10% dextran sulfate, 500 μg/ml tRNA, 10 mM dithiothreitol, 0.3 M NaCl, 10 mMTris, pH 8.0, and 1 mM EDTA, pH 8.0). The next day, the slides were washed with RNAse A (10 μg/mL Fisher Scientific, Pittsburgh, PA, USA) to remove any unbound RNA, and salinity was adjusted using decreasing concentrations of standard sodium citrate buffer (SSC). Sections were then dehydrated, dried, and apposed to Kodak BioMax-MR film (Fisher Scientific, Pittsburgh, PA, USA) along with [^14^C]-standards of known radioactivity (Amersham Bioscience, Buckinghamshire, England) where they were exposed at 4°C.

For darkfield images, slides were dipped in Kodak autoradiography NTB emulsion (VWR, West Chester, PA, USA) and exposed at 4°C. Slides were developed in Kodak D-19, fixed in Kodak Professional fixer (Fisher Scientific, Pittsburgh, PA, USA), counter-stained with Cresyl-Violet and cover slipped with DPX mounting medium (Fluka, Ronkonkoma, NY, USA).

### Analysis

A computer-based image analysis system (MCID, Imaging Research Inc., St. Catherine, Canada; now InterFocus Imaging Ltd., UK) was used to analyze ^35^S-hybridization signals from autoradiograms. For quantification of the hybridization signal, ^14^C-standards were used to construct a calibration curve comparing relative optical density (ROD) to radioactivity in nCi/g. Hybridization intensities was calculated by converting ROD for antisense probes into nCi/g as a measure of expression intensity, which was then adjusted for decay based on the ^35^S calibration date. Mean values of expression intensity and standard errors were calculated.

Microscopic analysis of the mRNA hybridization signals was performed using an Olympus BX1 microscope equipped for light- and dark-field microscopy. Images were taken with a DP7–1 digital camera (DP manager, Leeds Instruments, Irving, TX, USA).

## Results

### Adult Expression of Npas4 mRNA

Npas4 mRNA expression in adult mouse brain was mostly restricted to telencephalic structures (Figure 1). The highest hybridization intensity was detected in the AON, piriform cortex, frontal cortex, cingulate cortex, auditory cortex and hippocampus. Moderate expression was found in somatosensory and visual cortices. Low levels of expression were detected in the dorsal caudate putamen (CPu), amygdala and in retrosplenial cortex. Most areas of the diencephalon or mesencephalon exhibited no expression, with the exception of the arcuate and paraventricular nucleus (PVN) of the hypothalamus.

### Npas4 mRNA Expression During Postnatal Development

**Figure 2:**
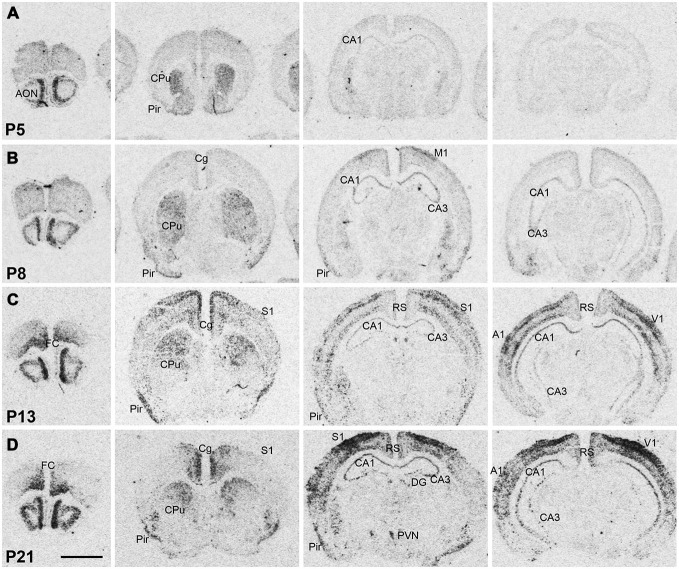
**Npas4 mRNA expression in postnatal mouse brains.** Autoradiographic images of Npas4 mRNA hybridization in coronal sections of the anterior olfactory nucleus (AON) and frontal cortex, caudate putamen (CPu), dorsal hippocampus, and ventral hippocampus at **(A)** postnatal day (P)5, **(B)** P8, **(C)** P13, **(D)** P21. Abbreviations: see Figure 1. Scale bar = 2 mm.

**Figure 3:**
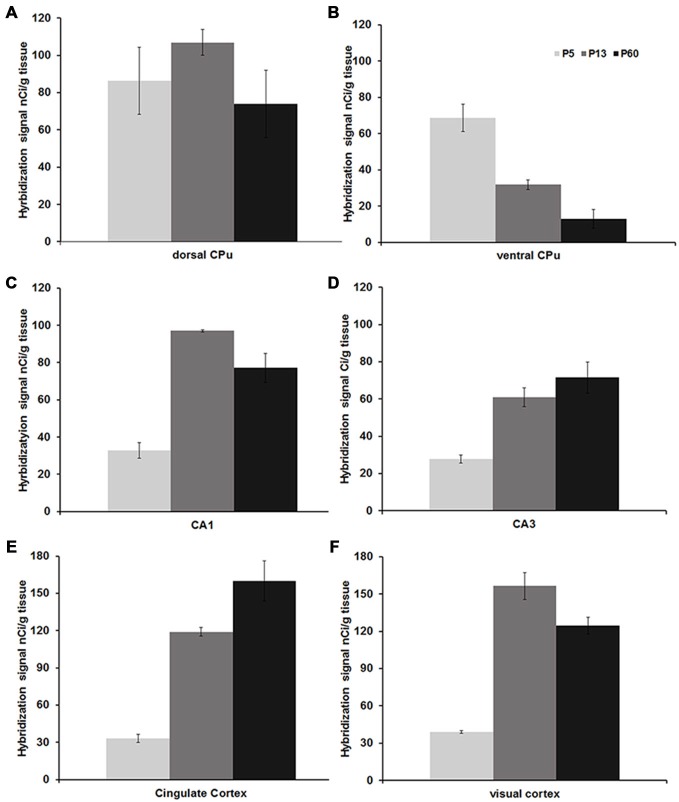
**Mean Npas4 hybridization intensities.** Expression in **(A)** dorsal caudate, **(B)** ventral caudate, **(C)** CA1 stratum pyramidale, **(D)** CA3 stratum pyramidale, **(E)** cingulate cortex, **(F)** primary visual cortex. Light gray bars = postnatal day (P)5, dark gray bars = P13, black bars = P60; *n* = 3. Error bars = standard error.

Npas4 expression was low in neonates (P1 and P3), and hybridization signals were only detected in the CPu and AON (data not shown). At P5, strong signal was located in the AON, where it remained high into adulthood, and moderate expression was found in the piriform cortex and CPu (Figure 2A). At the beginning of the second postnatal week (P8), low hybridization signal was also detected in a superficial layer of primary motor (M1) cortex, and in the pyramidal layer of dorsal and ventral hippocampus (Figure 2B). However, expression remained low in other cortical areas at P8. During the second and third postnatal week, signal intensities strongly increased in neocortex, cingulate cortex and in the pyramidal layer of CA1 and CA3 hippocampus, reaching adult levels of expression intensities (Figures 2C,D, 3). In the amygdala and septum, expression remained low. In general, areas that exhibited expression during postnatal development were the same that exhibited expression in adult brain areas. The exception was the CPu, where transient hybridization signal was found in ventral regions during early postnatal development. However, with increasing postnatal age, the expression became restricted to the dorsal CPu with little or no expression in ventral CPu (Figures 2, 3A,B).

### Darkfield Analysis Of Npas4 mRNA Expression

**Figure 4:**
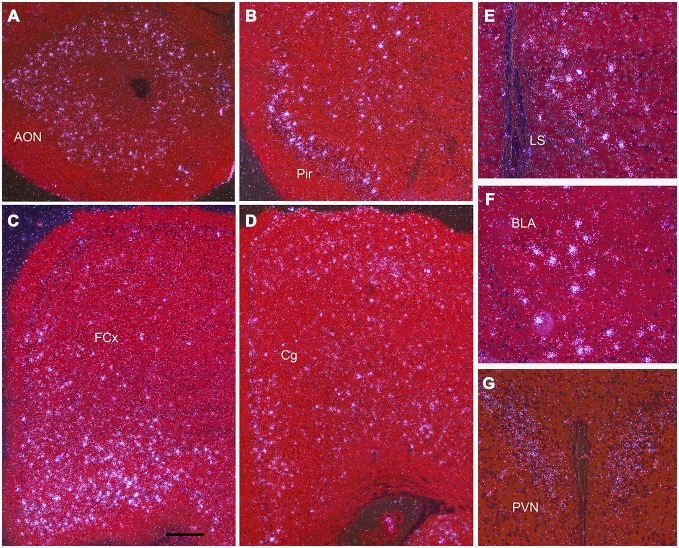
**Npas4 mRNA expression at postnatal day (P)21.** Darkfield photomicrographs of hybridization in **(A)** the anterior olfactory nucleus (AON), **(B)** pirifrom corex (Pir), **(C)** Frontal cortex (FCx), **(D)** cingulate cortex (Cg), **(E)** lateral septum (LS), **(F)** basolateral amygdala (BLA), **(G)** paraventricular nucleus (PVN). Scale bar = 250 μm in **(A—D)**, 100 μm in **(E—G)**.

**Figure 5:**
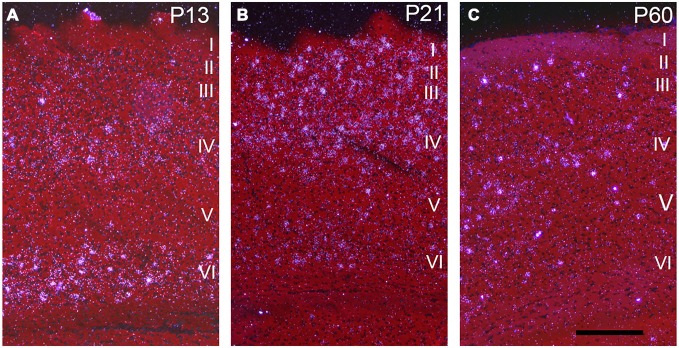
**Npas4 mRNA expression in primary visual cortex.** Darkfield photomicrographs of hybridization in coronal sections at **(A)** postnatal day P13, **(B)** P21 and **(C)** P60. The Roman numerals indicate the cortical layers in primary visual cortex. Scale bar = 250 μm.

Darkfield microscopy revealed that most Npas4 mRNA was located in scattered cells in forebrain structures (Figures 4A–F). At P21, Npas4 mRNA expression was detected in scattered cells in the lateral septum and basolateral amygdala (BLA; Figures 4E,F). In contrast, expression in the PVN was more homogeneous (Figure 4G). In P13 neocortex, the number of cells exhibiting Npas4 expression was highest in layer VI and IV as shown for primary visual cortex (Figure 5A). At P21, Npas4-expressing cells were found primarily in layers I-IV and VI, with only a few cells detected in layer V (Figure 5B). By P60, Npas4 mRNA expression was reduced to some scattered cells in layers II-VI (Figure 5C). However, in auditory cortex, expression remained more pronounced in adults.

**Figure 6:**
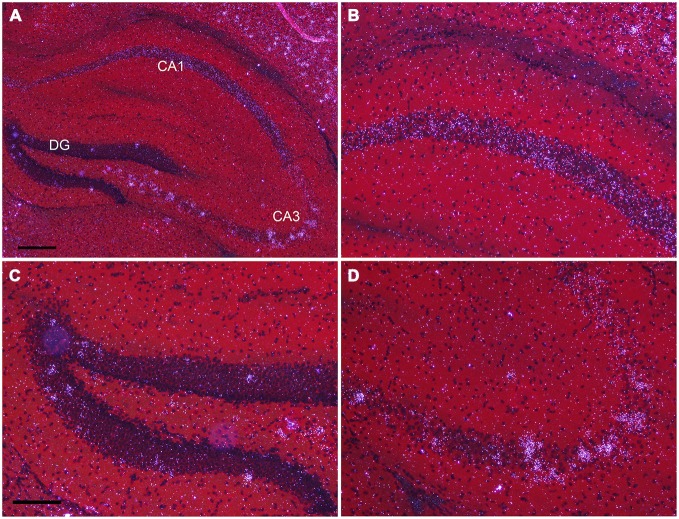
**Npas4 mRNA expression in dorsal hippocampus (A) Darkfield photomicrographs of hybridization in the dorsal hippocampus, (B) in the CA1, (C) in dentate gyrus, (D) in CA3 at postnatal day 21.** Scale bars: in **(A)** = 250 μm, in **(C)** = 125 μm (applies to **B—D)**.

In the hippocampus, NPas4 mRNA was detected in CA1, CA2, CA3 and DG with similar expression levels in dorsal and ventral hippocampus (Figure 6). Moderate NPas4 mRNA hybridization signal was evenly distributed in pyramidal cells of the stratum (s.) pyramidale in subfield CA1 and CA2 (Figure 6B), whereas in CA3 s. pyramidale, numerous individual cells with robust signal were found (Figure 6D). In the DG, NPas4 mRNA was found in a few cells located in the granule cell layer and in the hilus region (Figure 6C). Almost no expression was detected in cells located in s. oriens or radiatum of either CA1 or CA3, or in the molecular layer of the DG.

## Discussion

This study describes the expression pattern of the transcriptional regulator Npas4 in postnatal and adult male mouse brains using highly sensitive isotopic *in situ* hybridization with an Npas4 specific cRNA probe. In adults and during development Npas4 expression was almost exclusively found in areas of the telencephalon, including neocortex, hippocampus, basal ganglia and olfactory bulb, with some expression also detected in the septum and amygdala. In contrast, Npas4 expression in non-telencephalic brain areas including the diencephalon and mesencephalon was very low and restricted to the hypothalamic arcuate and PVN. In most of telencephalic areas, Npas4 mRNA expression started during the first and second postnatal week and increased in intensity with little change in spatial distribution compared to adults. The exception was the CPu, where Npas4 expression became increasingly restricted to the dorsal CPu.

### Expression of Npas4 in the Adult Brain

Previous studies using northern blot analysis had detected Npas4 mRNA (then called LE-PAS or Nxf) in frontal and entorhinal cortices, hippocampus and olfactory bulb (Moser et al., 2004). Studies using non-radioactive *in situ* hybridization found additional mRNA expression in hippocampus and neocortex (Lin et al., 2008; Moser et al., 2004). In addition to expression in hippocampus and neocortex, Npas4 mRNA transcripts were also detected in thalamus and striatum of adult rat brain (Ooe et al., 2004). To verify the distribution pattern of Npas4 mRNA in adult mouse brain, we used isotopic *in situ* hybridization with an antisense cRNA probe specific for Npas4, allowing for high spatial resolution and detection of mRNA transcripts in single cells (Winzer-Serhan et al., 1999). With this approach, we verified Npas4 expression in telencephalic areas including the olfactory system, neocortex, hippocampus and the amygdala, and confirmed striatal expression in mouse brain. In contrast to results from rat (Ooe et al., 2004), expression in the diencephalon was limited to the hypothalamic PVN and arcuate nucleus. This difference might be explained by differential expression of NPas4 mRNA between rodent species. However, in the unchallenged mouse brain used in this study, Npas4 mRNA was mostly expressed in regions of the telencephalon, and exhibits no or very limited expression in the diencephalon and mesencephalon.

NPas4 mRNA was not uniformly expressed in all neurons of the telencephalon, but was restricted to scattered cells which exhibited strong hybridization signal. The exceptions were the olfactory bulb and CA1 s. pyramidale, areas that exhibited strong or moderate expression in most neurons, which was not detected with the sense probe. The scattered distribution of cells with intense signal suggests that Npas4 expression may be localized in GABAergic neurons, in temporarily active excitatory neurons, or in both, but the identity of these cells still needs to be determined. Certain areas harbored more Npas4 positive neurons than others. For example, increased numbers were detected in frontal, cingulate and auditory cortices, which may suggest excitatory activity strong enough to maintain strong Npas4 expression. Particularly striking was the strong Npas4 hybridization signal in a subset of neurons located in the hippocampal CA3 s. pyramidale and in the granule layer of the DG. There is a sub-population of CA3 pyramidal cells which originate from early born glutamatergic neurons. These neurons are highly connected and are considered central nodes in the functional organization of hippocampal networks (Marissal et al., 2012). Thus, it is possible that these neurons are constitutively active and therefore, continuously express Npas4 mRNA. However, further studies need to verify the identity of these cells expressing high levels of Npas4 and to correlate neuronal activity to Npas4 expression levels under basal conditions in the mature male brain.

A previous report found expression of Npas4 in interneurons located in s. oriens and radiatum (Moser et al., 2004). However, in this study, there was very little or no expression in cells located in s. oriens and radiatum The reasons for this discrepancy is not clear, but since Npas4 is an immediate early gene that is easily induced within minutes by a variety of stimuli (Flood et al., 2004; Shamloo et al., 2006; Ramamoorthi et al., 2011), it is possible that differential handling and/or housing conditions prior to sacrificing the animals could account for this difference.

In addition to the expression in the hippocampus and neocortex, Npas4 expression was robust in the olfactory system, including olfactory bulb, AON and piriform cortex starting in neonates and persisting into adulthood. These areas have high GABAergic tone and high densities of GABAergic synapses which are strongly involved in the regulation of excitatory activity (Ribak et al., 1977). Npas4 might have an important role in the regulation of inhibitory synaptic plasticity, and in the maintenance of the excitatory/inhibitory balance in the olfactory system, an idea that is supported by recent findings that Npas4 expression is rapidly increased by odor presentation (Bepari et al., 2012). In addition, a functional role of Npas4 in the developing sensory olfactory system has recently been described (Yoshihara et al., 2014). Interestingly, in that study, the majority of Npas4 positive neurons represent GAD67 positive interneurons indicating a role in inhibitory synapse formation in inhibitory neurons.

### Developmental Expression of Npas4

Npas4 mRNA expression fully emerged during postnatal development. Prior to birth, Npas4 mRNA hybridization signal was very low, when evaluated in embryonic brain sections from gestational age 18 and 21 embryos (data not shown). There was a general pattern of increasing expression intensity during the first two postnatal weeks in telencephalic areas, which established the adult-like pattern. The olfactory system exhibited robust expression, shortly after birth, with other brain areas following thereafter. A similar expression pattern has been described in other vertebrate species including the developing zebrafish brain (Klarić et al., 2014) GABAergic and glutamatergic.

During late prenatal and early postnatal development, the first functional synapses formed in cortical structures are GABAergic, followed by glutamatergic synapses, which are initially silent during the neonatal period (Ben-Ari et al., 1997; Tyzio et al., 1999; Hennou et al., 2002; Gozlan and Ben-Ari, 2003; Voigt et al., 2005; Ben-Ari, 2006). Thus, early developmental GABAergic synaptogenesis takes places in the absence of excitatory glutamatergic synaptic activity, and we expected to see onset of Npas4 expression during the developmental period when GABAergic synapses are initially formed. However, in most cortical areas that was not the case. Instead, cortical Npas4 mRNA expression was very low in neonates, suggesting that the initial formation of GABAergic synapses is independent of Npas4 activity. This is in line with studies showing that Npas4 knockout mice develop normal inhibitory synapses (Lin et al., 2008). However, it remains to be seen if an excitatory stimulus could drive Npas4 expression in the immature brain, and thereby affect GABAergic synapse formation during the perinatal period.

In most neocortical areas, Npas4 mRNA expression strongly increased by the end of the second postnatal week, especially in layers IV and VI of sensory cortices (see Figure 2). It has been shown that induction of Npas4 mRNA depends on triad of NMDA receptor, AMPA receptor and voltage-gated calcium channel (VGCC) activation (Lin et al., 2008). This would suggest that mature glutamatergic synaptic transmission consisting of NMDA + AMPA receptor mediated components needs to be in place in order to effectively induce Npas4 mRNA expression. With increasing postnatal age, the relative NMDA/AMPA receptor ratio decreases, so that by the end of the second postnatal week mature NMDA + AMPA receptor-mediated excitatory synaptic transmission is established (Golshani and Jones, 1999; Brill and Huguenard, 2008). This coincides with increased activity of thalamocortical axonal projections relaying sensory information to the cortex by the end of the second postnatal week (Agmon and O’Dowd, 1992, Feldmeyer and Radnikow, 2009; Dorrn et al., 2010). This postnatal time point also represents a period of profound transformational changes in cortical synaptic network activities (Micheva and Beaulieu, 1997; Lendvai et al., 2000; Stern et al., 2001; Maravall et al., 2004), culminating in the functional maturation of large-scale cortical network activity around P13 in rodents (Quairiaux et al., 2011), which correlates with the sharp increase in Npas4 mRNA expression at P13 in sensory cortices detected in this study. Thus, although correlative, Npas4 mRNA expression seems to follow the maturation of excitatory cortical circuits, perhaps marking the beginning of activity-driven inhibitory synapse formation, which could explain the large increase in the density of inhibitory synapses at the beginning of the third postnatal week (De Felipe et al., 1997).

In the hippocampus, developmental Npas4 expression exhibits a similar pattern as seen in the cortex by temporally following the maturation of excitatory synapses. Comparable to neocortical development, GABAergic synapses appear first, followed by glutamatergic ones, and most excitatory pyramidal neurons are silent, and have no or a very small AMPA receptor component in the neonatal hippocampus, (Durand et al., 1996; Hsia et al., 1998; Tyzio et al., 1999; Hennou et al., 2002). The maturation of excitatory glutamatergic synaptic responses takes place in the hippocampus during the first postnatal week (Hsia et al., 1998; Leinekugel, 2003). Npas4 mRNA expression greatly increased between P5 and P8 in CA1 and CA3 s. pyramidale, supporting the notion that mature AMPA + NMDA receptor mediated excitatory transmission drives Npas4 mRNA expression. It remains to be determined if aberrant excitatory activity, perhaps caused by neonatal seizures, could induce Npas4 mRNA expression in the hippocampus at an earlier age and alter the formation of inhibitory synapses.

## Conclusion

Npas4 is a transcriptional regulator that could have a significant regulatory role in setting up cortical circuits and shaping the excitatory/inhibitory balance in the developing brain. Adult expression of Npas4 is restricted to telencephalic regions where scattered cells with high levels of expression are found. In the developing mouse brain, Npas4 mRNA distribution exhibits a similar spatial distribution pattern. The onset of Npas4 mRNA expression during the second and third postnatal week seems to correlate with the maturation of excitatory synapses from functionally silent into active ones as AMPA receptors become increasingly incorporated in postsynaptic membranes. It remains to be determined if an increase in neuronal excitatory activity is responsible for the increase in Npas4 expression during postnatal development. However, this information could serve as a starting point to explore the potential roles of Npas4 during development, which in turn could lend insight into some of the mechanisms of activity-dependent brain development.

## Author Contributions

JCD: conducted the experiments, generated a riboprobe specific for Npas4, analyzed the results, prepared the manuscript. GSS: conducted the experiments, helped in writing the manuscript. UHWS: Planned and oversaw the project, analyzed the results, prepared the manuscript.

## Conflict of Interest Statement

The authors declare that the research was conducted in the absence of any commercial or financial relationships that could be construed as a potential conflict of interest.

## Abbreviations

AON: anterior olfactory nucleus
BLA: basolateral amygdala
CPu: caudate putamen
Npas4: neuronal PAS domain-containing protein 4
P: postnatal day
VGCC: voltage-gated calcium channels.
